# Cellular v-ATPase is required for virion assembly compartment formation in human cytomegalovirus infection

**DOI:** 10.1098/rsob.160298

**Published:** 2017-11-01

**Authors:** Jonathan Pavelin, Dominique McCormick, Stephen Chiweshe, Saranya Ramachandran, Yao-Tang Lin, Finn Grey

**Affiliations:** Division of Infection and Immunity, The Roslin Institute, University of Edinburgh, Easter Bush, Midlothian EH25 9RG, UK

**Keywords:** human cytomegalovirus, v-ATPase, assembly and egress, herpesvirus, host virus interaction

## Abstract

Successful generation of virions from infected cells is a complex process requiring orchestrated regulation of host and viral genes. Cells infected with human cytomegalovirus (HCMV) undergo a dramatic reorganization of membrane organelles resulting in the formation of the virion assembly compartment, a process that is not fully understood. Here we show that acidification of vacuoles by the cellular v-ATPase is a crucial step in the formation of the virion assembly compartment and disruption of acidification results in mis-localization of virion components and a profound reduction in infectious virus levels. In addition, knockdown of ATP6V0C blocks the increase in nuclear size, normally associated with HCMV infection. Inhibition of the v-ATPase does not affect intracellular levels of viral DNA synthesis or gene expression, consistent with a defect in assembly and egress. These studies identify a novel host factor involved in virion production and a potential target for antiviral therapy.

## 1. Introduction

Herpesvirus assembly is a complex process involving viral and host factors [1,2]. In brief, viral DNA is synthesized in the nucleus and packaged into nucleocapsids. These particles then escape the nuclear lamina and bud through the inner nuclear membrane into the perinuclear space, acquiring a primary envelope in the process. This viral envelope then fuses with the outer nuclear membrane, allowing egress of the nucleocapsid into the cytoplasm. Once in the cytoplasm, tegument proteins assemble on the capsid, and secondary envelopment occurs as the virion buds into the virion assembly compartment (VAC) that is derived from the trans-Golgi network (TGN) and/or endoplasmic reticulum (ER) membranes containing the viral glycoproteins. The mature virions are then trafficked within these vesicles to the cell surface where they are released into the extracellular space.

The human cytomegalovirus lytic replication cycle is long, releasing very few particles until approximately 72 h post-infection (hpi), during which time dramatic morphological changes occur in the cell to facilitate viral assembly. Specifically, at these late times post-infection the cytoskeleton remodels [3], the nucleus increases in volume and changes in morphology (taking on a kidney shape) [4], and secretory vacuoles reorganize [3,5]. These secretory vacuoles redistribute to form a juxtanuclear cytoplasmic inclusion called the viral assembly compartment (VAC). This is the site where the majority of viral tegument proteins and host cellular proteins [6] assemble on the surface of the nucleocapsid before final envelopment occurs [3,7].

The HCMV VAC is derived from *trans*-Golgi network derived vacuoles, early endosomes, and vacuoles bearing markers of the ESCRT III machinery [3,5,8,9]. Despite re-localizing to form the VAC, markers for these compartments remain distinct, which suggests that different compartments may perform different roles [9]. However, the specific mechanics of assembly within these compartments is not well understood.

Until recently, the viral and host cellular factors that are involved in the biogenesis of the VAC have been poorly defined. Work by Das *et al.* using siRNAs against key HCMV early-late and late viral genes has identified several viral proteins that are required for the proper development of the VAC (UL48, UL94 and UL103) [10]. Bughio *et al.* have established that the UL133-138 locus is required for VAC formation specifically in endothelial cells [11]. In addition to these viral factors, several critical host genes have been identified that are essential in VAC biogenesis [12]. More recently multiple HCMV miRNAs have been shown to be important for VAC formation and virion production. The secretory pathway genes VAMP3, RAB5C, RAB11A, SNAP23 and CDC42 have been identified as targets of HCMV encoded microRNAs. Downregulation of these secretory pathway genes by miR-UL112-1, US5-1 and US5-2 facilitates the formation of the VAC [13]. Together, the current evidence indicates that the biogenesis of the VAC is an elaborate process regulated by the complex interplay of host cellular compartments, their associated proteins and viral gene products.

Previously we identified ATP6V0C, the enzymatic component of the vacuolar ATPase (v-ATPase) as an important host factor for HCMV replication [14]. Here, we show that the v-ATPase is required for correct VAC formation, and knockdown or inhibition of v-ATPase results in a profound and specific assembly and egress phenotype during HCMV infection.

## 2. Material and methods

### 2.1. Cells and viruses

Normal human dermal fibroblast (NHDF) cells (Clonetics) were cultured in Dulbecco's modified Eagle's medium supplemented with 10% fetal bovine serum (FBS) and penicillin-streptomycin-L-glutamine (PSG). HCMV strain AD169 was obtained from the American Type Culture Collection (Rockville, MD). TB40/E-GFP was obtained from Dr Goodrum [15]. All HCMV strains were grown on primary fibroblast cells following infection at low multiplicity of infection (MOI). Virus stocks were isolated from cleared supernatant over 10% sorbitol gradients as described previously [16].

### 2.2. Small RNA transfections

Cells were transfected with small RNAs using RNAiMAX lipofectamine reagent (Life Technologies) according to manufacturer guidelines with the following modifications. Fibroblast cells were double transfected with 20 pmol (40 nM final concentration) of small RNA per 24 wells 8 h apart. Control cells were transfected with a non-targeting negative control siRNA (cat. 1027310, Life Technologies). ATP6V0C (S80) and ATP6V1H (s28403) siRNA were purchased from Life technologies. Confirmation of gene knockdown was ascertained at an RNA level using qRT-PCR with specific primer probe sets against ATP6V1H and ATP6V0C (Invitrogen). The efficacy of knockdown was in excess of 95% up to 7 days post-transfection (data not shown). Cell viability was established using CellTiter-Blue (Promega) according to the manufacturer's instruction.

### 2.3. Western blot analysis

Human primary fibroblast cells were grown in 10% FBS supplemented DMEM before infection at a multiplicity of 3 with AD169 or TB40/E-GFP. At 72 hpi, cells were harvested using SDS sample loading buffer. 30 µl of protein sample were loaded and proteins were probed using primary antibodies to TGN46, (cat. PA5-23068, Pierce), EEA1 (cat. ab2900, Abcam), pp28 (cat. CH19, Santa Cruz) and gB (cat. 2F12, Abcam) according to the manufacturer's guidelines. Protein loading was normalized to GAPDH (Sigma). IR800 or IR680 dye conjugated anti-rabbit IgG and anti-mouse IgG secondary antibodies were purchased from LiCor. Blots were imaged using infrared fluorescence of appropriately tagged secondary antibodies and quantified using a LiCOR Odyssey scanner and software.

### 2.4. Viral growth curve analysis

Viral growth curve analyses were performed as described in [16]. NHDF cells were transfected as described above. At 72 h post transfection with siRNAs, cells were infected at an MOI of 1. At 24 hpi, cells were washed three times with PBS and overlaid with fresh media. Infected cells were harvested, scraped into media, at 24 hpi, and at every subsequent 24-h time-point. Samples were snap frozen at −80°C to lyse the cells. These lysates were then serially diluted, and used to infect sub-confluent NHDFs. At 24 hpi, cells were overlaid with 0.5% carboxymethyl-cellulose (CMC) diluted in DMEM +10% FBS+PSG. At 168 hpi, the CMC was removed, monolayers were stained with toluidine blue and plaques were counted.

### 2.5. Chloroquine treatment

NHDFs were seeded at 60–80% confluence in 24-well plates. Twenty-four hours prior to infection, cells were overlaid with DMEM +10% FBS and PSG containing chloroquine to a final concentration of 1.25, 2.5, 5, 10 and 25 µM. Cells were infected with TB40/E-GFP at an MOI of 1. At 24 hpi, the infectious inoculum was removed, and cells were washed three times with PBS. Cells were then overlaid with media containing chloroquine at the indicated concentrations. Cells were harvested 7 days post-infection by scraping into the media and frozen at −80°C. Virus levels were determined by plaque assay as previously described. Cell viability was established using CellTiter-Blue (Promega) according to the manufacturer's instruction.

### 2.6. RT-PCR analysis

Total RNA was harvested using Trizol with concentrations and RNA quality determined by nano-drop spectrophotometer analysis. 100 ng of total RNA was DNAse treated (Promega) then reverse transcribed using high capacity cDNA reverse transcription kit (ABI). Real time PCR was performed using gene specific primer probe sets from ABI on a Rotor gene 3000 (Corbet Research). Specific primer probe sequences were kindly provided by Lauren Hook. IE86, UL83 and gH primer and probes were synthesized by Life Technologies. The sequences for the primers for these assays are as follows: gH (UL75); TTGCTAGCTCATCCGCACC (primer 1), AAGAGACGCGTAAGGCGTTC (primer 2), CAGCGACCTGTACACACCCTGTTCCAGTAG (probe), HCMV IE86; ATGTCCTGGCAGAACTCGGT (primer 1), GCTGCAAGAGTGGGTTGTCA (primer 2), CCAGTAGCACCGGCCCCACG (probe), HCMV UL83; TGGAGAACGTGTCGGTCAAC (primer 1), GGATGTTCAGCATCTTGAGCG (primer 2), AGCCAGGAGCCCATGTCGATCTATGTGTAC (probe). Relative expression levels were determined by delta delta Ct calculation with levels corrected to GAPDH levels.

### 2.7. Viral genome copy analysis

Infected cells were harvested at designated time-points and applied to DNEasy blood and tissue kit columns (Qiagen). DNA was extracted following the manufacturer's protocol. IE86, UL83 and gH primer and probes were synthesized by Life Technologies. A primer probe set against gB was used to quantitate viral DNA genome levels. For supernatant viral genomes, virus was isolated by ultracentrifugation over a sorbitol cushion as previously described [16]. The viral pellet was resupended in DNase buffer and split into two aliquots, one of which was treated for 1 h at 37°C with Turbo DNase (Ambion). DNA was then isolated by phenol chloroform purification and PCR analysis performed as above.

### 2.8. Immunofluorescence and confocal microscopy

NHDF's were seeded for siRNA transfection, and double-transfected as previously described. At 72 h post-transfection, cells were infected with AD169 at an MOI of 1. At 144 hpi, coverslips were washed with PBS, and fixed for 15 min with freshly prepared 4% paraformaldehyde in Dulbecco's PBS then permeabilised for 5 min (DPBS+3% FBS+0.2% TX100. Coverslips were then blocked for 1 h at room temperature (DPBS+3% FBS+0.5% Tween 20), before the addition of primary antibodies to TGN46, (cat. PA5-23068, Pierce), EEA1 (cat. ab2900, Abcam), pp28 (cat. CH19, Santa Cruz), gB (cat. 2F12, Abcam) according to manufacturers guidelines. Coverslips were incubated with primary antibodies overnight at 4°C. Coverslips were washed 3 times with DPBS+3% FBS before the addition of secondary fluorescent antibodies (AlexaFluor anti-mouse 647 and anti-rabbit 488, Life Technologies). Coverslips were incubated for 1 h at room temperature, and washed three times with DPBS+3% FBS. Finally, coverslips were nuclear stained using 1 : 1000 DAPI before mounting to slides using 5 µl Prolong Gold anti-fade reagent (Life Technologies).

Images were acquired using a Zeiss LSM 710 confocal microscope in accordance with the manufacturer's instructions. Images were compiled and analysed using Fiji open source image processing software [17]. Parallel images were generated using human serum as a blocking agent to rule out possible cross reactivity with the viral Fc receptor that has been reported for some Rabbit polyclonal antibodies. Day four time point samples and cells treated with chloroquine were blocked with human serum (1%) prior to staining.

### 2.9. Electron microscopy

Fibroblast cells were cultured, transfected with siRNA, and infected as specified above, and were fixed in 3% glutaraldehyde buffer at 120 hpi then washed in three 10 min changes of 0.1 M sodium cacodylate. Specimens were then post-fixed in 1% osmium tetroxide in 0.1 M sodium cacodylate for 45 min, then washed in three 10 min changes of 0.1 M sodium cacodylate buffer. These samples were then dehydrated in 50%, 70%, 90% and 100% ethanol (X3) for 15 min each, then in two 10 min changes in propylene oxide. Samples were then embedded in TAAB 812 resin. Sections 1 µm thick were cut on a Leica Ultracut ultramicrotome, stained with Toluidine Blue, and viewed in a light microscope to select suitable areas for investigation. Ultrathin sections 60 nm thick were cut from selected areas, stained in uranyl acetate and lead citrate, then viewed in a JEOL JEM-1400 Plus TEM. Representative images were collected on a GATAN OneView camera.

### 2.10. Data presentation

Data are presented throughout as a mean of multiple biological replicates (*N* = *x*), and error is displayed as the standard deviation from the mean. Student *t*-test was used to determine significance where indicated. For nuclear area analysis, a two-way ANOVA test with replication was used.

## 3. Results

### 3.1. Knockdown of ATP6V0C causes an assembly and egress defect in HCMV infected cells

In a previous siRNA screening study we demonstrated that knockdown of ATP6V0C prior to infection with GFP expressing HCMV resulted in a modest reduction in reporter gene expression compared with negative control transfected cells, but a pronounced reduction in infectious virus production [14]. The differential effects on reporter gene expression and virus production suggest a defect in virus assembly and egress. To further investigate this phenomenon we measured the production of infectious virus by plaque assays and compared this to the levels of viral genome amplification in infected cells. Production of infectious virus and viral genome copy number was independently measured in cell lysates and virus from the supernatant of infected cells, thereby independently measuring intracellular versus secreted virus levels.

**Figure 1.**
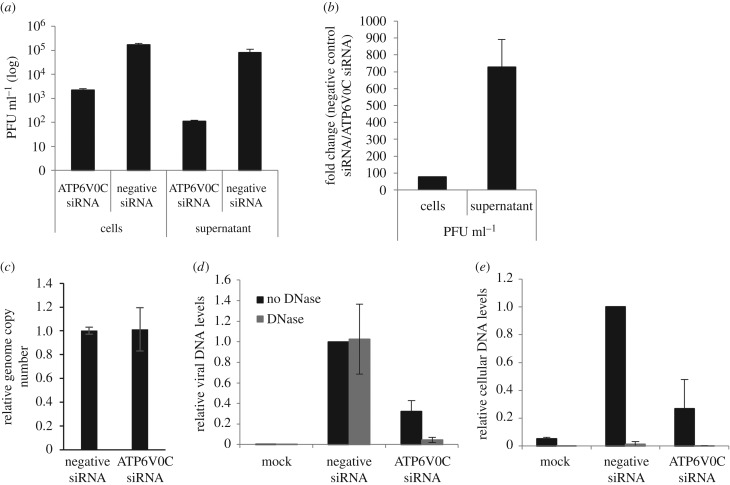
Knockdown of ATP6V0C has greater effect on supernatant virus than cell associated virus. Fibroblast cells were transfected with siRNA against ATP6V0C or a control siRNA and infected with TB40/E-GFP at an MOI of 1 and cells and supernatant collected 7 days post-infection. (*a*) Infectious cell associated and supernatant virus levels were determined by dilution plaque assay following ATP6V0C knockdown. (*b*) Effects of ATP6V0C knockdown on infectious supernatant virus levels are greater than effects on cell associated virus. (*c*) Cell associated viral genome levels were equivalent between control cells and ATP6V0C knockdown cells at 7 days post-infection (*d*) Supernatant virion genome levels were determined by qPCR. Supernatant was collected from cells 7 days post infection. Virions were isolated from supernatant by ultracentrifugation then treated with DNase to degrade non-virion associated viral DNA. Primers against HCMV gB were used to determine viral genome levels and primers to GAPDH (*e*) were used to confirm successful degradation of non-protected DNA (*n* = 2).

The titre of cell-associated virus from ATP6V0C siRNA transfected fibroblasts was almost 2-log lower than that observed in negative control cells 7 days post-infection (figure 1*a*). However, in the supernatant virus from ATP6V0C siRNA transfected cells, the titre was approximately 3-log lower than the negative control (figure 1*a*). These data are consistent with previous results from plaque assays on cell associated and supernatant virus combined, which showed a 2–3-log decrease in viral titres [14]. However, the data shown in figure 1*b* demonstrate that there was an approximately 80-fold reduction in virus titre in cell associated virus and an approximately 700-fold reduction in virus titre in supernatant in ATP6V0C siRNA transfected fibroblast cells compared with the negative control cells, illustrating an order of magnitude difference when comparing cell-associated with supernatant virus following ATP6V0C knockdown. While infectious virus titres were substantially reduced, viral genome levels were equivalent at 7 days post-infection, suggesting the defect in virus production is downstream of virus genome amplification (figure 1*c*). The reduction in infectious virus is not due to cytotoxic effects as there was little difference in the viability of control or ATP6V0C knockdown cells over the course of the 7-day infection (electronic supplementary material, figure S1).

The dramatic loss in plaque forming units in the supernatant could be due to a failure in production of cell free infectious particles or a defect leading to the generation of non-infectious particles. To investigate this, virion associated genomes were measured in supernatant from infected control or ATP6V0C knockdown cells. To differentiate between virion genomes and free viral DNA released from lysed cells, virions were isolated by ultracentrifugation then treated with DNase. Virion-associated genomes would be protected from DNase, whereas free viral DNA would be degraded. While the majority of detectable viral DNA from control cells was resistant to DNase treatment, indicating association with virions, DNA detected from ATP6V0C knockdown cells was sensitive to DNase treatment, suggesting that the majority of viral DNA detected is not associated with virions (figure 1*d*). Cellular DNA, measured using GAPDH primers, was not protected in supernatant from control or ATP6V0C knockdown cells, confirming successful DNase treatment (figure 1*e*). Higher levels of GAPDH DNA were detected in the supernatant of infected cells compared with uninfected cells, probably due to cytotoxic effects and cell death caused by virus infection. Release of cellular DNA was lower in ATP6V0C knockdown cells, possibly reflecting higher levels of cell viability at late time points (electronic supplementary material, figure S1). This suggests a loss of supernatant viral particles when ATP6V0C is knocked down, although the production of non-infectious particles that lack viral DNA, or unstable particles that lose structural integrity once released from the cell cannot be ruled out.

**Figure 2.**
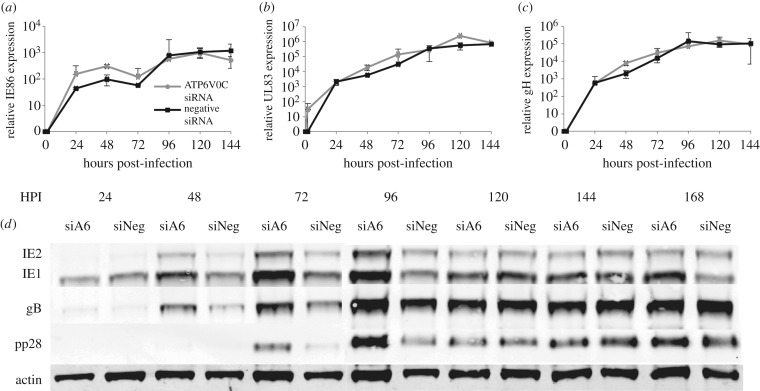
Knockdown of ATP6V0C has no effect on levels of representative viral immediate early or late transcripts. Fibroblast cells were transfected with ATP6V0C siRNA or a control siRNA and infected 48 hpi. Total RNA was harvested at indicated time points. Levels (*a*) of IE86, (*b*) UL83 and (*c*) UL75 were determine by qRT-PCR using specific primer probe assays (*n* = 2). (*d*) Cell-associated viral protein levels were determined by western blot analysis at the indicated time points post-infection from control cells (siNeg) or cells knocked down for ATP6V0C (siA6).

To determine whether knockdown of ATP6V0C causes a defect in viral gene expression, quantitative RT-PCR was performed on RNA extracted from infected cells transfected with ATP6V0C siRNA or a negative control siRNA. Relative transcript levels were determined for the immediate early transcript IE86 and two late transcripts UL83 (pp65) and UL75 (gH) (figure 2*a–c*). Knockdown of ATP6V0C did not result in a significant reduction in the expression of viral transcripts. However, an increase in viral transcription was observed at early time points in ATP6V0C knockdown cells. A corresponding increase in viral protein expression at early times in cells knocked down for ATP6V0C was also observed (figure 2*d*). It is currently unclear why knockdown of ATP6V0C results in increased viral gene expression at early time points. However viral gene expression from 120 hpi is similar in control and knockdown cells, suggesting that the defect in virus replication caused by knockdown of ATP6V0C occurs down stream of viral entry, DNA amplification and viral gene expression, consistent with a defect in assembly and egress.

### 3.2. ATP6V0C is not required for generation of viral capsids in the nucleus

**Figure 3.**
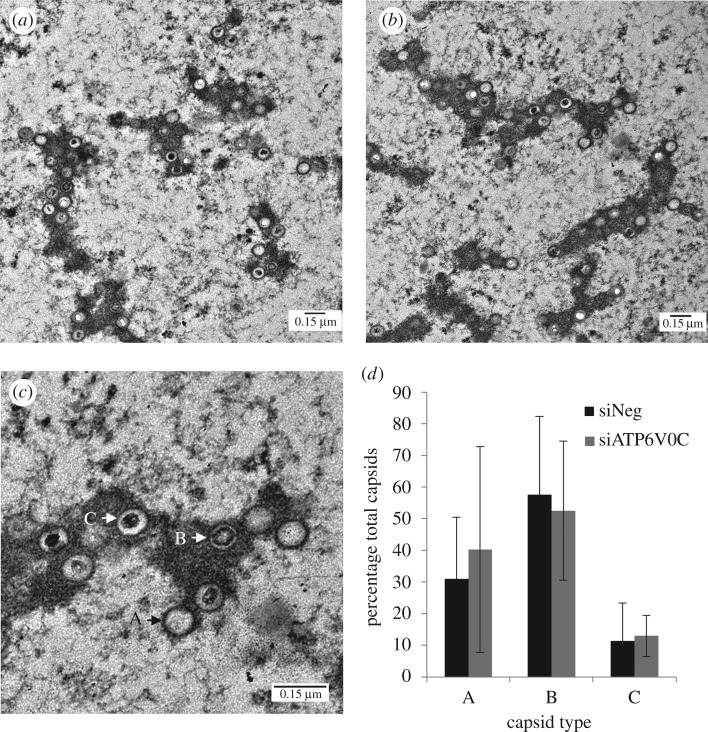
ATP6V0C is not required for generation of viral capsids in the nucleus. To directly analyse the effects of ATP6V0C knockdown on intracellular viral particle formation, infected cells were imaged by transmission electron microscopy at 120 hpi. Fibroblast cells were transfected with (*a*) ATP6V0C siRNA or (*b*) negative control siRNA and infected with HCMV (AD169). (*c*) Enlarged image from (*b*) showing three capsid types: A—empty capsid, B—capsid with scaffold, C—DNA containing capsid. (*d*) Manual counting of images revealed no significant difference in nuclear capsid formation following ATP6V0C knockdown. Nine individual frames from three independent cells were counted for each condition. A total of 508 capsid particles were counted for control cells and 516 particles counted for ATP6V0C knockdown cells.

To further define at which stage virus production is blocked after ATP6V0C knockdown we analysed infected cells by electron microscopy (EM). At 120 hpi, viral capsids can clearly be observed within the nucleus of infected ATP6V0C knockdown cells (figure 3*a*) and in control siRNA transfected cells (figure 3*b*), suggesting no defect at this stage of infection. Different stages of capsid formation can also be discerned by EM analysis corresponding to empty capsids, capsids containing scaffold proteins, and capsids containing viral genomes (figure 3*c*) [12]. Manual counting of 508 and 516 total individual capsids from negative control and ATP6V0C knockdown cells, respectively, did not support statistically significant differences in the numbers of each type of capsid between control cells and ATP6V0C knockdown cells (figure 3*d*). While EM analysis can be a relatively insensitive method for characterizing defects in virus replication due to sampling of thin subsections of the infected cell, these data suggest no gross defect in virus capsid formation following ATP6V0C knockdown and that the defect in virus production probably occurs downstream of capsid assembly in the nucleus.

### 3.3. ATP6V0C is required for HCMV virion assembly compartment biogenesis

Following infection with HCMV, host-cellular endocytic and exocytic membrane compartments undergo dramatic reorganization, with the formation of the VAC late in infection [3]. Given the role of the cellular v-ATPase in organelle acidification and trafficking, we asked whether knockdown of ATP6V0C could be disrupting viral driven membrane reorganization and VAC formation.

**Figure 4.**
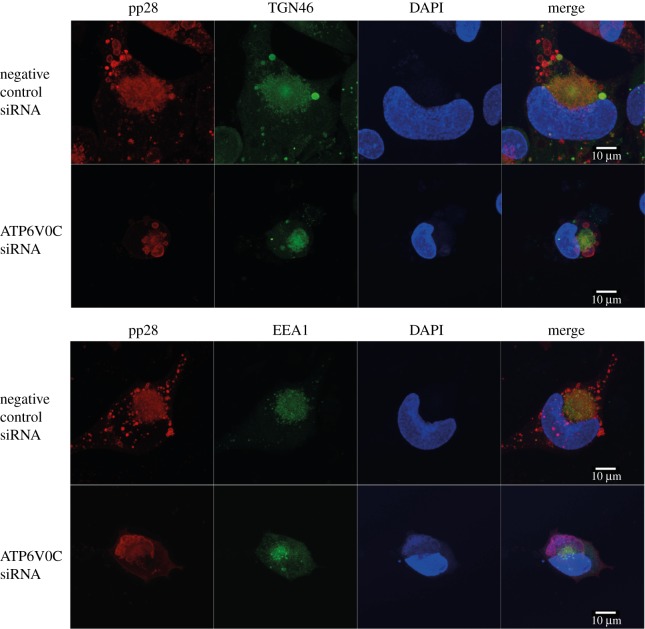
ATP6V0C is required for HCMV virion assembly compartment biogenesis. Fibroblast cells were transfected with ATP6V0C siRNA or negative control siRNA and at 72 h post-transfection were infected with AD169. At 144 hpi cells were fixed, permeabilised and stained for early endosomes or trans-Golgi vacuoles, (EEA1:green or TGN46:green), viral tegument protein (pp28:red) and nuclei (DAPI:blue). Images were acquired on a Zeiss LSM710 confocal microscope. Images presented here are maximum-intensity projections compiled from multiple 0.33 µm slices through the *z*-axis.

In order to visualize the HCMV VAC, immunofluorescence microscopy was performed to observe the host cellular markers, trans-Golgi network 46 (TGN46) and early endosome antigen 1 (EEA1) with the virion structural protein pp28. These are well-characterised markers of the HCMV VAC. TGN46 is a type I integral membrane protein that localizes to the trans-Golgi network and is thought to play a role in exocytic vesicle formation. EEA1 localizes to the early endosomal sorting compartment involved in endocytosis. In control cells, host cellular proteins TGN46 and EEA1 colocalize with the viral tegument protein pp28 in a region adjacent to an enlarged, kidney shape nucleus (figure 4). This staining is diffuse, lacking any obvious boundaries or puncta, and is consistent in localization and appearance with previous reports of the HCMV VAC [3,5,8–11]. Staining of untransfected cells also showed the same defined colocalization of cellular and viral markers (data not shown).

In cells transfected with siRNA against ATP6V0C, there were several observable morphogenic differences to control cells. The nuclei were smaller and the typical kidney shape of an HCMV infected cell was not apparent (figure 4). The host cellular markers of the VAC appear to localize to a similar position within the cell, but staining of TGN46 is less diffuse than that observed within a typical VAC, and the staining of EEA1 has clear boundaries, indicative of a large vesicular compartment. Most strikingly, pp28 does not colocalize with the host cellular markers of the VAC (figure 4).

**Figure 5.**
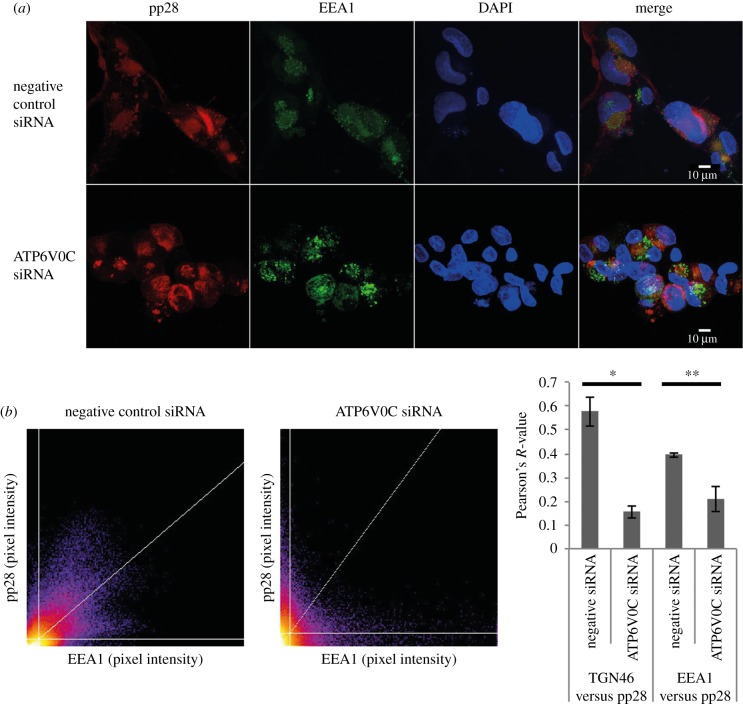
Loss of VAC due to ATP6V0C1 knockdown is quantifiable and significant. (*a*) Fibroblast cells were transfected with ATP6V0C siRNA or negative control siRNA and at 72 h post-transfection were infected with AD169. At 144 hpi cells were fixed, permeabilised and stained for early endosomes or trans-Golgi vacuoles, (EEA1:green), viral tegument protein (pp28:red) and nuclei (DAPI:blue). Images represent single slices through the *Z*-axis. (*b*) Representative scatter-plot showing average pixel signal intensity in red (pp28) and green (EEA1) channels from multi-cell images (*n* = 16 for ATP6V0C and *n* = 9 for negative siRNA). Individual images in the Z-field were analysed using Fiji image analysis software. (*c*) Pearson's *R*-value for colocalization of TGN46 or EEA1 and pp28 in ATP6V0C or negative control siRNA transfected fibroblast cells (*n* = 20). *p-value < 0.05; **p-value < 0.01.

Images from a wider field of view demonstrate that this defect is representative (figure 5*a*). Importantly, these qualitative observations are quantifiable. Scatter plots of pixel intensity from a multi-cell field demonstrated that staining for TGN46 and pp28 overlapped extensively in negative control cells, but did not for cells transfected with an siRNA against ATP6V0C (figure 5*b*). Colocalization analyses of TGN46, EEA1 and pp28 were performed, with Pearson's R scores calculated (1 = convergent 0 = divergent). The frequency of TGN46 and pp28 staining convergence was high in negative control cells (*R* = 0.58), and low in ATP6V0C siRNA transfected cells (*R* = 0.16). Similarly, EEA1 and pp28 staining convergence was high in negative control cells (*R* = 0.41) and low in ATP6V0C siRNA transfected cells (*R* = 0.17) (figure 5*c*). Staining of earlier time points (96 hpi) also showed distinct staining of TGN46 in ATP6V0C knockdown cells, as did an additional cellular marker associated with the VAC, GM130. In both cases a loss of staining around the edge of central pp28 staining was observed, indicating a defective organization of the VAC (electronic supplementary material, figure S2*a*,*b*).

**Figure 6.**
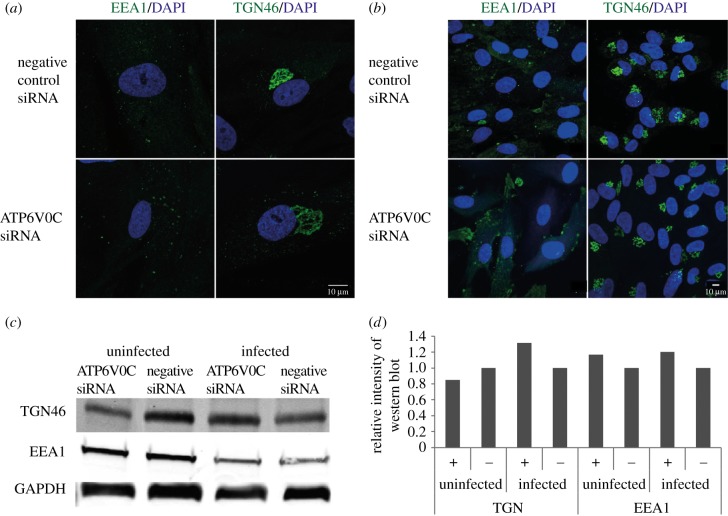
Failure of VAC formation following AT6V0C knockdown is not due to gross defects in cellular membrane organization prior to infection. (*a*) Fibroblast cells were transfected with ATP6V0C siRNA or negative control siRNA. At 72 h post-transfection cells were fixed, permeabilised, and stained for early endosomes or trans-Golgi vacuoles. Images are maximum-intensity projections compiled from multiple 0.33 µM slices through the *z*-axis. (*b*) Wide field view of fibroblast cells from (*a*). Image is a single optical slice. (*c*) Western blot analyses of ATP6V0C siRNA and negative control and transfected fibroblast cells against markers of trans-Golgi vacuoles (TGN46), early endosomes (EEA1). Infected cells were harvested at 72 hpi. (*d*) Graph shows quantification of representative western shown in C.+ = ATP6V0C siRNA transfected fibroblast, − = negative control siRNA.

To examine whether the defect in VAC formation was due to a possible gross disruption of the secretory apparatus as a result of ATP6V0C knockdown, the distribution of TGN46 and EEA1 was analysed by immunofluorescence microscopy, and the abundance of these proteins was analysed by western blot analysis in uninfected cells. The distribution of both TGN46 and EEA1 was similar in uninfected fibroblast cells transfected with ATP6V0C siRNA when compared with a negative control (figure 6*a*,*b*). While this does not rule out defects in organelle function it does suggest that the organelles are structurally intact prior to infection. By 6 days post-transfection some differences were detectable in the staining pattern of the cellular marker GM130, perhaps unsurprisingly showing some defects following long-term knockdown of ATP6V0C (electronic supplementary material, figure S3). The protein abundance of TGN46 and EEA1 were similar in uninfected ATP6V0C siRNA and negative control siRNA transfected fibroblast cells 48 h post-transfection and infected cells, 72 hpi (figure 6*c*,*d*).

### 3.4. Knockdown of ATP6V0C blocks HCMV associated increase in nuclear area

**Figure 7.**
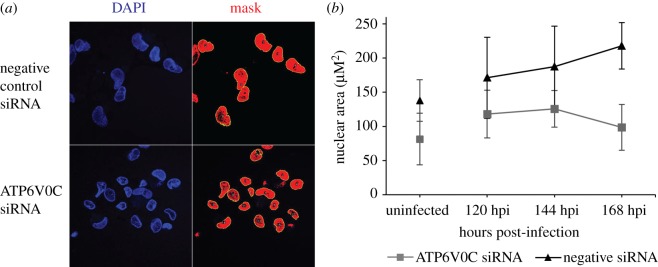
ATP6V0C knockdown disrupts changes in nuclear size. (*a*) Representative image showing nuclei in ATP6V0C and negative control siRNA transfected fibroblast cells infected with HCMV (AD169). Using Fiji imaging software, masks were drawn around nuclei and nuclear area was calculated. (*b*) Nuclear areas in ATP6V0C and negative control siRNA transfected fibroblast cells infected with HCMV (AD169) and mock-infected fibroblast cells. Quantification based on minimum 12 cells per condition per time point. Difference in cross-sectional nuclear area between control cells and ATP6V0C knockdown cells was significant based on two-way ANOVA analysis with repetitions *p* ≤ 0.01.

In addition to major reorganizations of cellular membranes, infection with HCMV causes a substantial increase in nuclear size [4]. However, an increase in nuclear area fails to occur following infection of fibroblast cells transfected with ATP6V0C siRNA. Using Fiji imaging software, the nuclear area of cells transfected with ATP6V0C siRNA were quantified, and shown to be smaller than those transfected with negative control siRNA (figure 7*a*,*b*). The mean nuclear area of cells transfected with ATP6V0C siRNA and infected with HCMV (AD169) was 55% smaller than negative control siRNA transfected cells at 168 HPI (figure 7*b*). Previous publications have observed a similar failure in nuclear enlargement associated with loss in virion assembly compartment formation and have suggested that the processes are intimately involved [18–21]. This provides further evidence that the cellular V-ATPase is required for the correct formation of the human cytomegalovirus assembly and egress cellular infrastructure.

### 3.5. v-ATPase acidification activity is required for efficient virus production

The v-ATPase is a membrane-spanning, multi-domain ion pump that is responsible for the regulation of pH in membrane-bound organelles throughout the cell. These channels consist of a luminal V_1_ domain, which is responsible for the hydrolysis of ATP, and the trans-membrane V_O_ domain, which forms the proton channel. ATP6V0C is a critical component of the V_O_ domain [22]. The establishment of a low intraluminal pH plays a crucial role within the endocytic and secretory pathways. There have also been several observations in a variety of contexts that the V_O_ domain of the v-ATPase may have membrane fusion activity that is independent of its role in the acidification of vacuoles. In mice, *C. elegans* and *Drosophila*, the V_O_ domain has been shown to interact with VAMP-2, syntaxin-1 and Ca^2+^ release channels to directly catalyse the mixing of two lipid bilayers [23–27].

**Figure 8.**
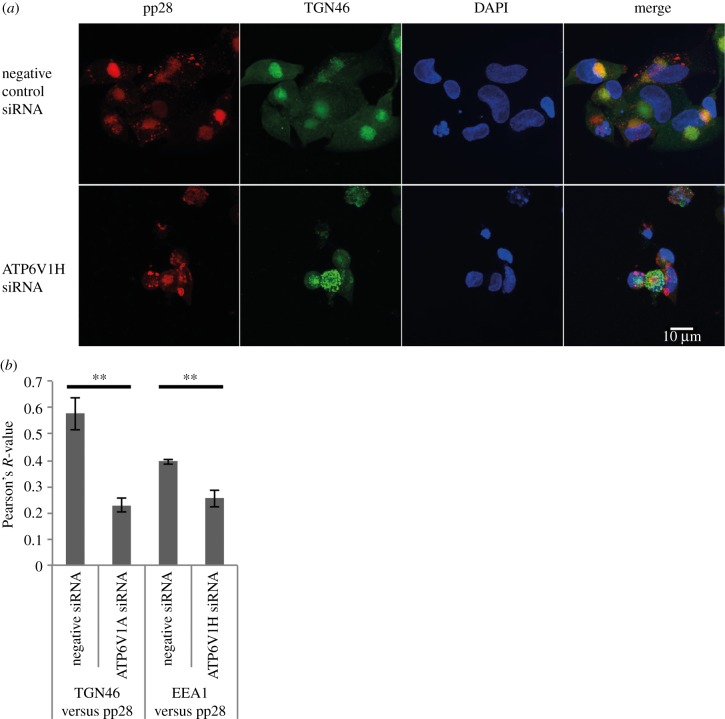
Disruption of V-ATPase complex results in loss of VAC formation. (*a*) Immunofluorescence microscopy in AD169 infected fibroblast cells transfected with ATP6V1H siRNA and stained for early endosomes (EEA1/green), viral tegument protein (pp28/red) and nuclei (DAPI/blue). Images represent single slices through the *z*-axis. (*b*) Pearson's *R*-value for colocalization of TGN46 or EEA1 and pp28 in ATP6V1H or negative control siRNA transfected fibroblast cells (*n* = 20). **p-value < 0.01.

To determine whether the assembly and egress phenotype caused by ATP6V0C knockdown was due to a block in vesicle acidification and disruption of the v-ATPase complex, another critical component of the v-ATPase, ATP6V1H, was knocked down using siRNA. ATP6V1H is the regulatory H subunit of the V1 domain of v-ATPase required for metabolism of ATP by the v-ATPase complex and therefore necessary for function, but it is distinct from the V_O_ domain. Figure 8 demonstrates that siRNA knockdown of ATP6V1H results in the same failure of VAC formation as observed following ATP6V0C knockdown. Therefore, disruption of the ATPase, rather than an independent function of ATP6V0C, appears to be responsible for the assembly and egress phenotype.

**Figure 9.**
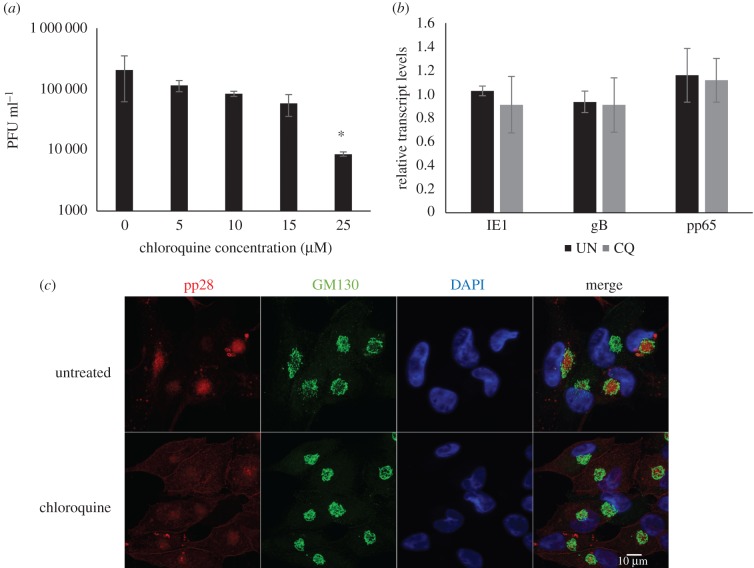
Chloroquine inhibits HCMV virus production and VAC formation. (*a*) Fibroblast cell were pre-treated 24 h before infection with the indicated concentrations of chloroquine. Following infection with TB40/E-GFP supernatant was harvested 7 days post-infection and virus levels determined by serial dilution plaque analysis. (*n* = 4, error bars indicate standard deviation, *p-value < 0.05). (*b*) Total RNA was extracted from control cells and cells treated as described above with 25 mM chloroquine (CQ), to determine viral transcript levels by qRT-PCR. Transcript levels were normalized to GAPDH and compared to untreated sample (UN) (*n* = 2, error bars indicate standard deviation). (*c*) Fibroblast cells were treated with 25 mM of chloroquine then infected with AD169 at an MOI of 1. Cells were fixed 96 hpi and analysed by immunofluorescence for viral pp28 (red), cellular GM130 (green) and nuclear stain DAPI (blue).

To confirm that inhibition of infectious virus production is due to a block in vacuolar acidification, virus replication was measured following treatment of cells with chloroquine. Chloroquine indirectly blocks the acidification of vacuoles. It freely diffuses through cells, but upon reaching an acidic compartment it undergoes a change of conformation that renders it impermeable, causing it to accumulate in these compartments [28]. This acts to buffer any further change in pH in these vacuoles. Fibroblast cells were incubated in media containing chloroquine 24 h prior to and during infection with the TB40/E-GFP strain of HCMV [15]. Inhibition of acidification of vacuoles within the cell with chloroquine had a dose dependent inhibitory effect on the replication of TB40/E-GFP (figure 9), and this was in the absence of any cytotoxic effects (electronic supplementary material, figure S4). Similar to ATP6V0C knockdown, viral transcription was relatively unaffected by chloroquine treatment at 7 days post-infection (figure 9*b*). Immunofluorescence staining of cells treated with chloroquine also showed defective organization of the VAC at 96 h post infection. Treatment with chloroquine resulted in a loss of GM130 staining at the periphery of the VAC, with GM130 staining showing a more compacted arrangement (figure 9*c*). In addition the nuclei in chloroquine treated infected cells are noticeably smaller (figure 9*c*).

## 4. Discussion

Here, we show that knockdown of ATP6V0C results in a profound assembly and egress phenotype that corresponds to a failure in VAC formation. The failure in VAC formation and virion production is due to a block in acidification of vacuoles linked to the role of ATP6V0C in the v-ATPase complex. While infectious supernatant virus was substantially reduced, viral DNA amplification and gene expression were not significantly impacted, consistent with an assembly and egress phenotype. Nuclear capsid formation was also not disrupted, suggesting a downstream block in virus production. However, virion-associated DNA levels were substantially reduced in the supernatant and there was a clear loss in cellular membrane and nuclear reorganization required for the formation of the virion assembly compartment.

The vATPases are multiprotein complexes that are responsible for regulating vacuolar pH via the transport of H+ ions across membranes [22]. The establishment of pH gradients between the cytosol and membrane bound organelles is fundamental to membrane trafficking pathways in the cell. Acidification of early endosomes allows for the release of ligand from internalized receptors, budding of multivesicular bodies is dependent on an acidic environment, while lysosomal acidification is required for the activation of degradative enzymes [29].

Within the context of VAC formation, the loss of function phenotype that is observed when ATP6V0C is knocked down is particularly intriguing. We observe that secretory and endocytic markers that normally colocalize with the viral assembly compartment fail to reorganize during infection when ATP6V0C is knocked-down using siRNAs. It may be that the failure of these compartments to reorganize results in a failure in VAC formation or causes a critical and deleterious change in VAC composition. We also observe that this effect is dependent on infection, with secretory marker localization and abundance not affected when ATP6V0C is knocked down in uninfected cells.

Further characterization of the viral particles produced may provide important clues as to the role of vacuolar acidification in virion assembly and egress. If defects in supernatant virions are subtle, for example, loss of specific viral glycoproteins or tegument proteins, this may point to a specific transport pathway associated with delivery of viral proteins to the site of assembly. However if gross defects are found, then failure of vacuole acidification may be linked to a more general failure of virus assembly.

We previously demonstrated that ATP6V0C is a target of the HCMV miRNA miR-US25-1 [14]. Given the effect ATP6V0C knockdown has on virus production this seems counterintuitive. It is possible that targeting ATP6V0C could be a mechanism of restricting virus production during latent infection. However, regulation of ATP6V0C by miR-US25-1 may not restrict virus production, as significant inhibition of ATP6V0C levels would not occur until late in infection, whereas the phenotype observed here was caused by knockdown of ATP6V0C before virus infection. Knockdown by miR-US25-1 may also be less robust compared with siRNA knockdown, resulting in different phenotypic outcomes. Irrespective of the role of miR-US25-1 targeting, ATP6V0C and the v-ATPase complex is clearly an important host factor in the assembly and egress of HCMV.

## Supplementary Material

Knockdown of ATP6V0C does not result in reduced cell viability

## Supplementary Material

Knockdown of ATP6V0C disrupts VAC formation at 96 hpi

## Supplementary Material

GM130 staining in uninfected cells six days post infection

## Supplementary Material

Chloroquine treatment is well tolerated in infected fibroblast cells

## Data accessibility

All data have been accessible within the manuscript or in the electronic supplementary material.
