# Supplementary figures and images for: Cellular v-ATPase is required for virion assembly compartment formation in human cytomegalovirus infection
Source: Open Biol. 2017 Nov 1;7(11):160298. doi: 10.1098/rsob.160298 (PMC5717334; doi:10.1098/rsob.160298)

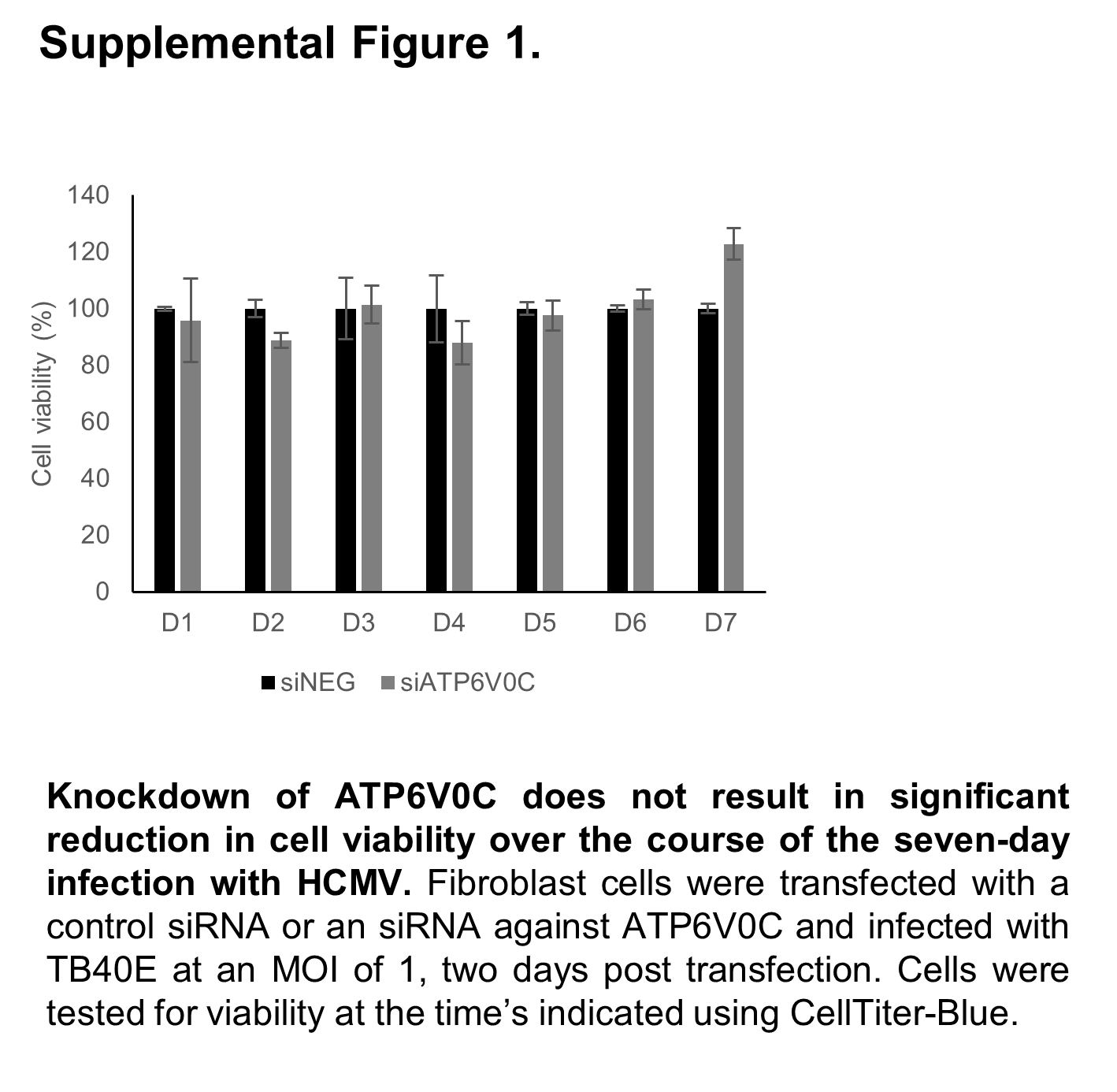

Supplement: Knockdown of ATP6V0C does not result in reduced cell viability [file rsob160298supp1.tif]

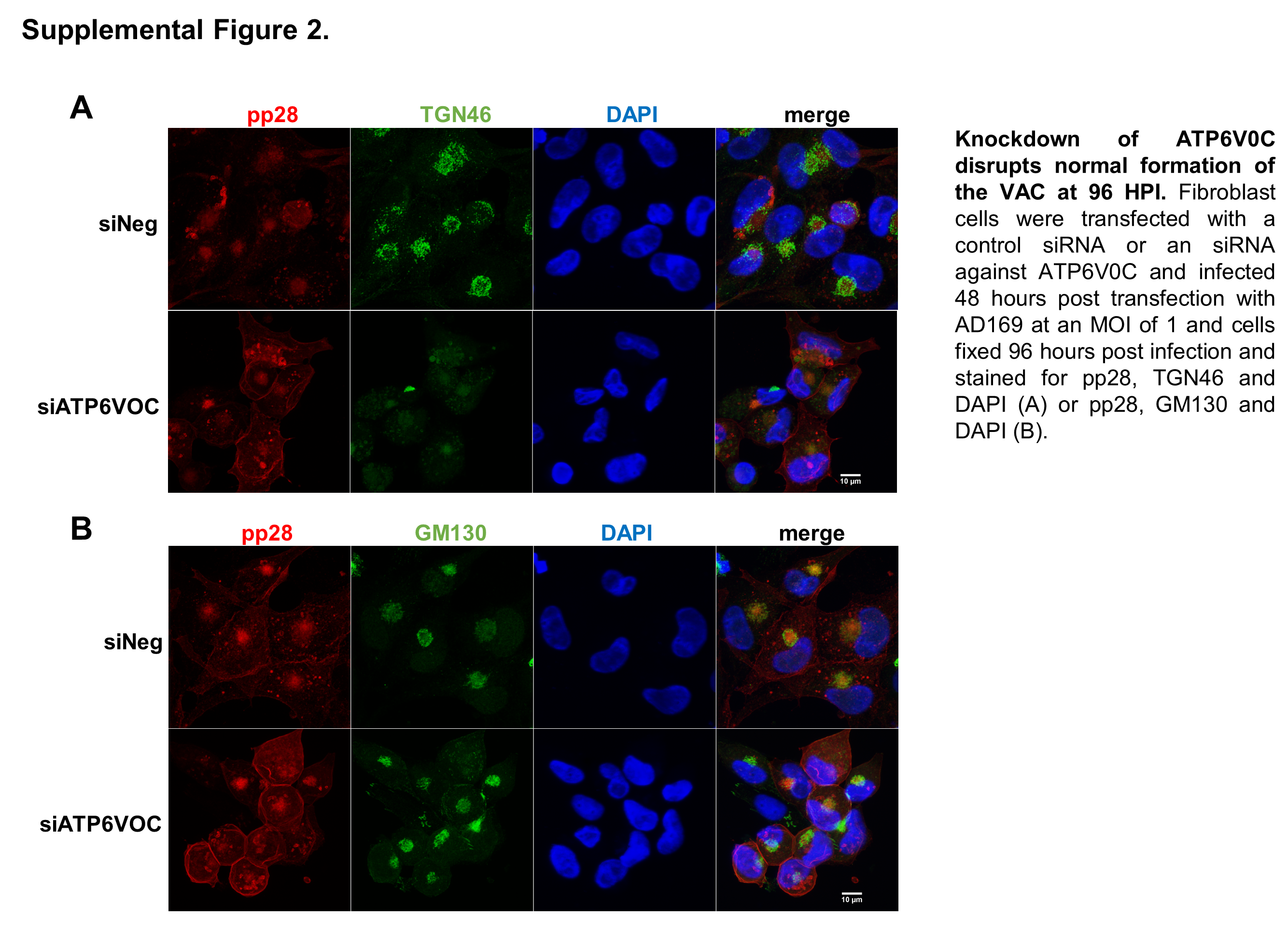

Supplement: Knockdown of ATP6V0C disrupts VAC formation at 96 hpi [file rsob160298supp2.tif]

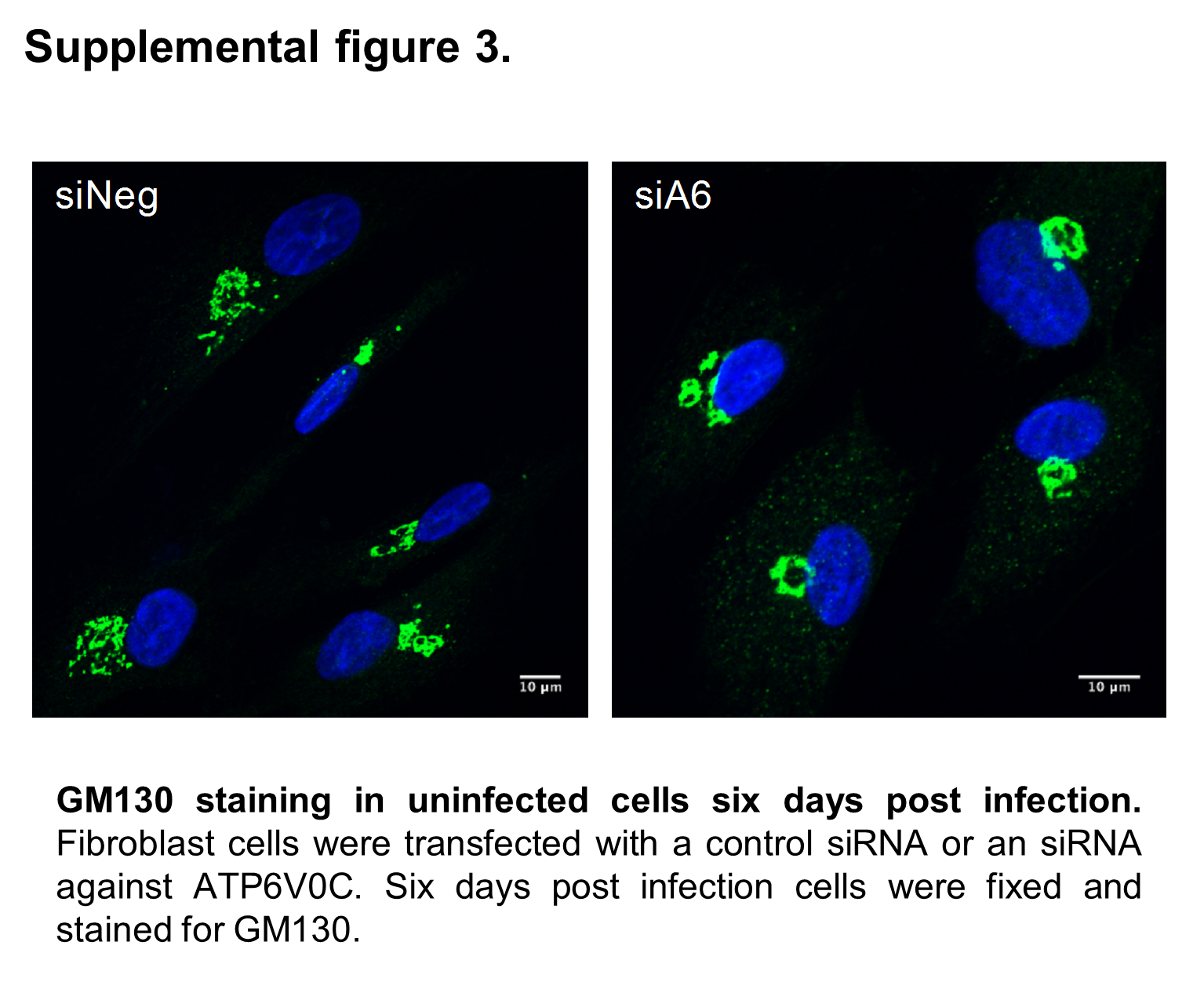

Supplement: GM130 staining in uninfected cells six days post infection [file rsob160298supp3.tif]

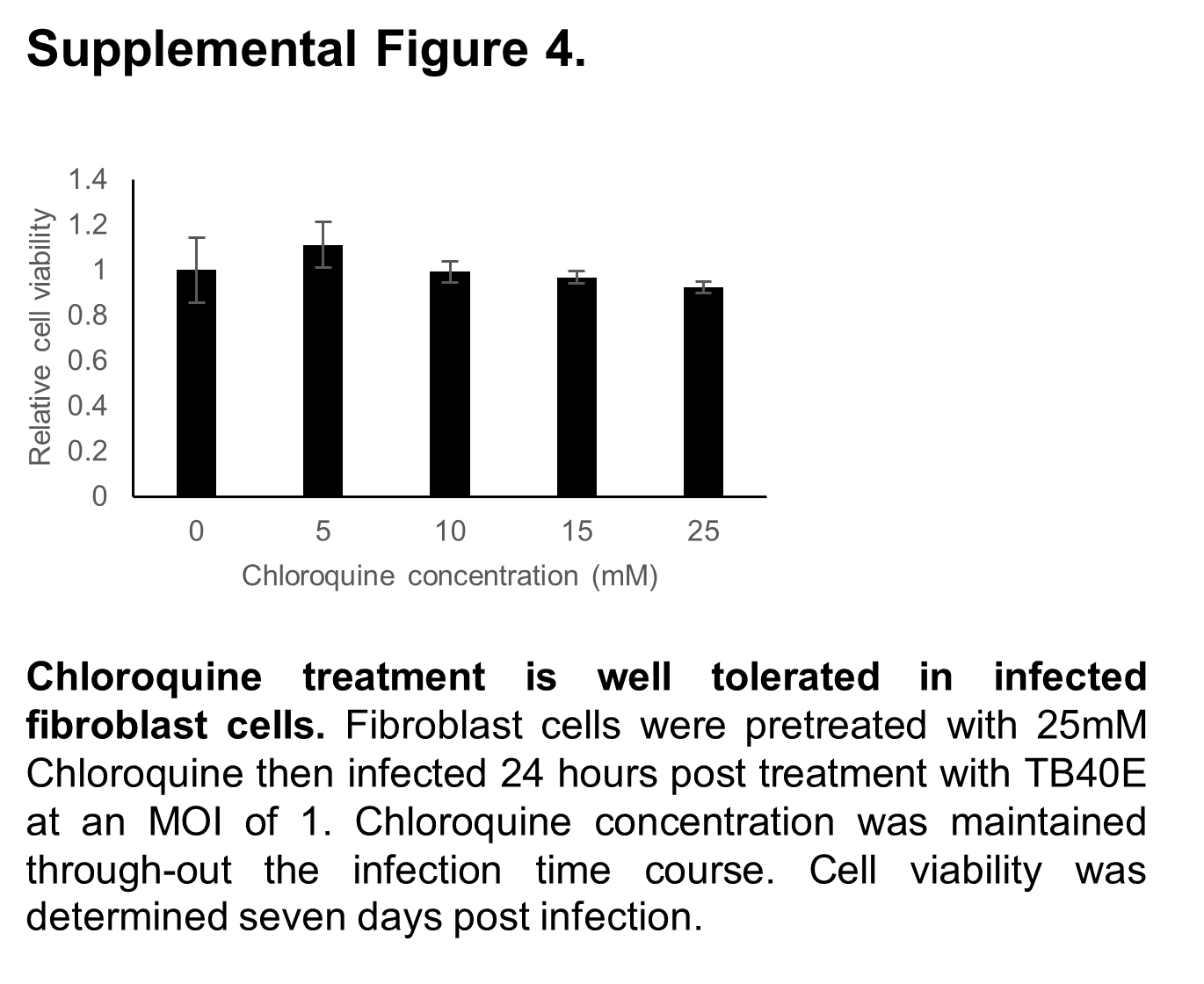

Supplement: Chloroquine treatment is well tolerated in infected fibroblast cells [file rsob160298supp4.tif]
